# Anti-Hepatoma Compound Determination by the Method of Spectrum Effect Relationship, Component Knock-Out, and UPLC-MS^2^ in *Scheflera heptaphylla* (L.)Frodin Harms and Its Mechanism

**DOI:** 10.3389/fphar.2020.01342

**Published:** 2020-09-09

**Authors:** Xuqiang Liu, Nan Jiang, Xiaoqing Xu, Cunyu Liu, Zhenhua Liu, Yan Zhang, Wenyi Kang

**Affiliations:** ^1^National R & D Center for Edible Fungus Processing Technology, Henan University, Kaifeng, China; ^2^School of Biomedical Sciences, Huaqiao University, Xiamen, China; ^3^Joint International Research Laboratory of Food & Medicine Resource Function, Henan Province, Henan University, Kaifeng, China; ^4^Hebei Food Inspection and Research Institute, Shijiazhuang, China; ^5^College of Forensic Medicine, Hebei Medical University, Shijiazhuang, China

**Keywords:** *S. octophylla*, spectrum-effect relationship, active ingredient, Huh7, mechanism

## Abstract

*Scheflera heptaphylla* (L.)Frodin, a kind of Traditional Chinese Medicine, is commonly used in anti-inflammatory, analgesic, anti-viral, anti-tumor, and hemostasis. This study aimed to determine the anti-hepatoma components and its mechanism from the leaves of *S. heptaphylla*. The spectrum-effect relationships were analyzed by the method of Partial least squares, indicating that P1, P2, and P10 were positively correlated to inhibitory activity of Huh7 cells. Whereas others were negatively correlated. The technologies of component knock-out and UPLC-MS^2^ were used to determine compounds as 3,4-Dicaffeoylquinic acid (P6), 3,5-Dicaffeoylquinic acid (P7), 3*α*-Hydroxy-lup-20(29)-ene-23,28-dioic acid (P10, named Compound A). The results forecasted that Compound A had the best correlation with inhibitory activity. The effects of Compound A on the activities of human hepatoma cells (Huh7, SMMC-7721, HepG 2) and normal hepatocytes (L0-2, Chang liver) were evaluated. Cell apoptosis was observed with inverted microscope and flow cytometer. In addition, the proteins, related to apoptosis, were detected by Western blot. The results showed that Compound A (400 nM) could significantly inhibit the activity of three hepatoma cells (*P* < 0.001) with slight toxicity to normal hepatocytes, and the IC_50_ values were 285.3 and 315.1 nM, respectively, which were consistent with the prediction of spectrum-effect relationships. After treatment with Compound A, the number of hepatoma cells decreased significantly. And the apoptosis rate of Huh7 cells increased significantly (*P* < 0.001) in Compound A (200, 400 nM) groups, SMMC-7721 and HepG 2 were directly necrotic. Compound A groups could significantly improve the level of intracellular reactive oxygen species (ROS) (*P* < 0.05, *P* < 0.001) in Huh7 with no effect on normal hepatocytes. The content of apoptotic protein (Bax and Bim) in mitochondria was significantly increased in Compound A groups (*P* < 0.001). On the contrary, the content of anti-apoptotic protein (Bcl-xL and Mcl-1) decreased significantly (*P* < 0.001). These results demonstrated that Compound A was the main anti-hepatoma active component in the *S. heptaphylla* leaves. It achieved the effect of promoting apoptosis of Huh7 cells by regulating the levels of ROS and Bcl-2 family protein in mitochondrial apoptosis pathway.

## Introduction

Hepatocellular carcinoma (HCC) is one of the most common malignant tumors in the world, with high morbidity and mortality (Ally et al., 2017; Fukuda et al., 2017; Yang et al., 2017; Yang et al., 2019; Wu et al., 2020). Primary liver cancer is the second leading cause of death in the world, of which 70% to 85% are HCC. The incidence of liver cancer in China ranks first worldwide (LV et al., 2015; Yang and Hou, 2016; Zhu and Liu, 2018).

According to statistics, the incidence of liver cancer is still growing rapidly, and China is a “Large country of liver cancer.” At present, the clinical treatment of liver cancer includes surgical treatment and non-operative treatment, and mainly choose the non-operative chemotherapy to improve the quality of life and prolong the life of patients (Julie et al., 2018). However, chemotherapeutic drugs could produce many side effects, such as myelosuppression, decreased immune function, organ damage, hair loss and so on, which significantly reduce the quality of life in patients. Ethnic drugs with wide efficacy and rich resources are widely used in the treatment of tumor, Such as *Eucommia* ulmoides Oliver, *Poria cocos* (Schw.) Wolf, and *Schisandra* chinensis (Turcz.) Baill (Li, 2011; Gui, 2012; Qian et al., 2019), which has the advantages of multi-target, multi-effect, little side effect, safety and effectiveness (Zhang et al., 2019). It could not only inhibit and kill tumor cells, but also has less toxicity to normal tissues and cells of the body. At the same time, it also can regulate the immune function of the body and improve clinical symptoms and signs (Wang and Hou, 2013). However, Traditional Chinses Medicine (TCM) is a whole concept in the process of treating diseases, it is difficult to determine the exact therapeutic components of TCM, which greatly restricts the application of TCM in clinical treatment of diseases.

*Scheflera heptaphylla* (L.)Frodin is commonly used as a hepatoprotective drug in Southeast Asia and has a significant anti-liver cancer effect. However, the anti-hepatoma effect of *S. heptaphylla* is not scientifically evaluated, and its active substances and mechanism are not clear. So, the anti-liver cancer active components of *S. heptaphylla* were investigated by the rapid method of “spectrum-effect relationship” (Wang et al., 2013), “knock-out technique,” and high-resolution mass spectrometry.

In this manuscript, the fingerprint of *S. heptaphylla* leaves was established by high performance liquid chromatography (HPLC) method, and the anti-hepatoma activity of 10 batches of extracts was evaluated *in vitro*. 10 quantitative chromatographic peaks were selected to analyze the correlation between the spectral efficacy and the efficacy data, and the spectral efficiency analysis model was established (Zeng et al., 2015). The peaks of anti-liver cancer active compounds were screened by spectrum-effect correlation, the target components were knocked out by knock-out technology (Gong et al., 2015), and the structures of the target components were identified by high resolution mass spectrometry. The effect and mechanism on anti-hepatoma of the target component was evaluated.

## Results

### Quantitative Determination of Chromatographic Peaks

Spectrum-effect relationship method was used to circumvent the weaknesses of the fingerprint technique and foster the strengths in TCM (Xu et al., 2014). The study of spectrum-effect relationship can reveal the material basis of the therapeutic values of TCM. This method has been widely applied in the researches of pharmacodynamic components, origins, harvest times, processing methods and batches of TCM (Hu et al., 2013; Zheng et al., 2014; Fang et al., 2016). Therefore, in order to accurately determine the material basis of their effects on anti-hepatoma activity, 10 batches of *S. heptaphylla* samples were selected to establish the fingerprint.

**Figure 1:**
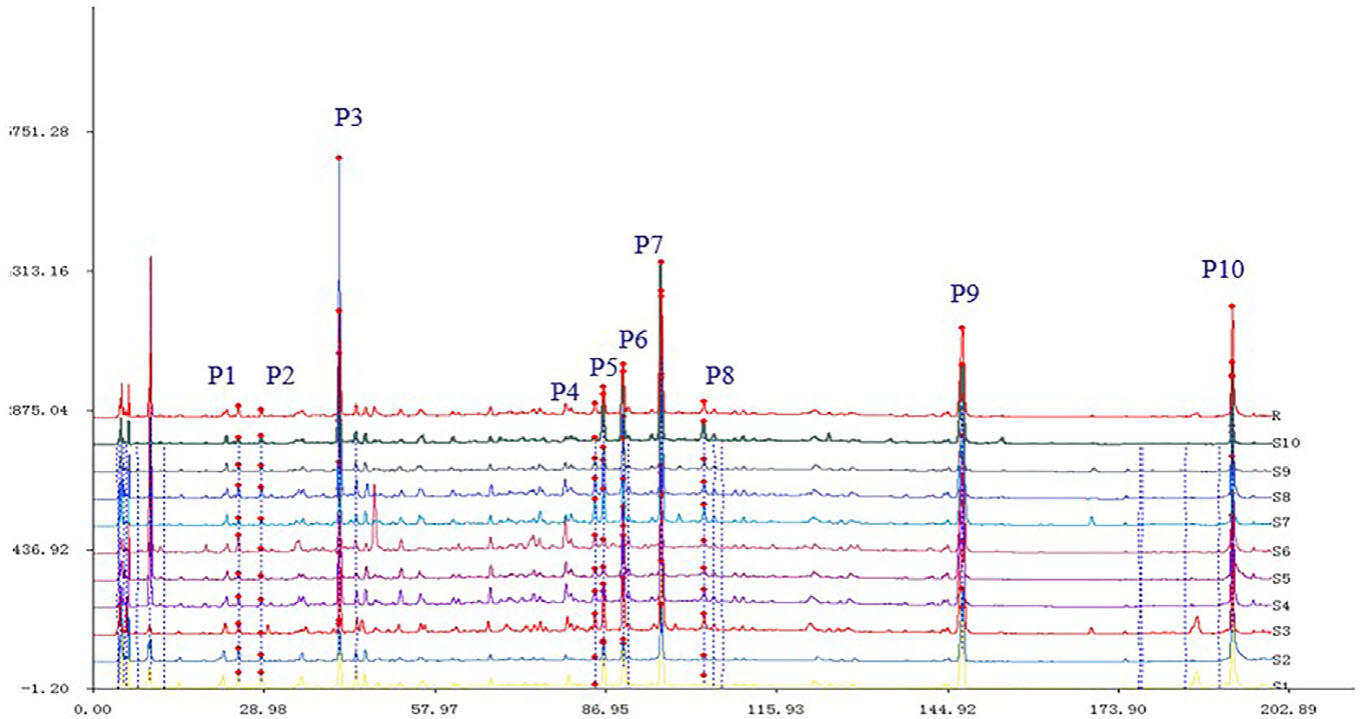
The matching chromatograms characterized by HPLC.

The chromatogram of *S. heptaphylla* ethanol extract was used as the reference spectrum, and the time window was set at 0.5. The chromatographic peaks with relatively high content and good separation in 10 batches of samples were selected for multi-point correction, and the average method was used to generate the control map by the software “Evaluation system of similarity of chromatographic fingerprint of traditional Chinese Medicine version 2004 1.0 A”. 10 common peaks out of the 10 feature maps were selected, as shown in **Figure 1**. The chromatographic peak matching data of *S. heptaphylla* leaves were shown in **Table 1** and the similarity data were shown in **Table 2**.

**Table 1:** The relative retention times and characteristic peak areas measured by HPLC (*n* = 10).

No.	Rt (min)	S1	S2	S3	S4	S5	S6	S7	S8	S9	S10
P1	24.651	4150363	3544788	2966786	2559433	1691232	4429168	2094106	3250961	1684673	1686005
P2	28.563	4685151	2096663	767523.3	2084688	1047507	1142884	1723123	2888822	1412197	2226924
P3	41.755	18577750	10369660	23167590	49942160	24822840	16189230	17097740	73437960	13291750	24401140
P4	85.11	592701.2	1457146	7990373	4320132	3009498	4750857	8561796	4586575	3546717	1556045
P5	86.568	13953340	5962813	14479290	5941413	3732601	4019646	10818650	10113450	6630262	13898410
P6	89.858	13179900	6474944	18474240	19940080	20712890	6278698	13510380	22922260	9318214	20823120
P7	96.315	38493880	20329800	64912050	39361480	23221780	16586590	48771280	57299430	29297410	55631030
P8	103.588	4301443	2003645	10780220	4782403	3473666	3193451	6878667	5054586	3843094	7659356
P9	147.446	32274540	30767130	53029030	52233150	54448330	53133420	51860980	48339290	31147070	37918510
P10	193.201	37645150	39480010	31020010	37507360	36340180	38904470	22636290	37593440	20405930	17547740

**Table 2:** The similarity degree of characteristic peak areas (*n* = 10).

No.	S1	S2	S3	S4	S5	S6	S7	S8	S9	S10	Reference fingerprint
S1	1	0.93	0.895	0.876	0.82	0.61	0.9	0.859	0.935	0.893	0.94
S2	0.93	1	0.805	0.855	0.857	0.681	0.847	0.782	0.902	0.778	0.907
S3	0.895	0.805	1	0.862	0.783	0.554	0.931	0.844	0.931	0.929	0.926
S4	0.876	0.855	0.862	1	0.913	0.704	0.886	0.947	0.911	0.877	0.966
S5	0.82	0.857	0.783	0.913	1	0.879	0.837	0.805	0.864	0.772	0.933
S6	0.61	0.681	0.554	0.704	0.879	1	0.604	0.544	0.631	0.52	0.743
S7	0.9	0.847	0.931	0.886	0.837	0.604	1	0.863	0.974	0.956	0.952
S8	0.859	0.782	0.844	0.947	0.805	0.544	0.863	1	0.892	0.899	0.922
S9	0.935	0.902	0.931	0.911	0.864	0.631	0.974	0.892	1	0.949	0.972
S10	0.893	0.778	0.929	0.877	0.772	0.52	0.956	0.899	0.949	1	0.929
Reference fingerprint	0.94	0.907	0.926	0.966	0.933	0.743	0.952	0.922	0.972	0.929	1

### Effect of Ethanol Extract From *S. heptaphylla* on the Viability of Huh7 Hepatoma Cells

**Figure 2:**
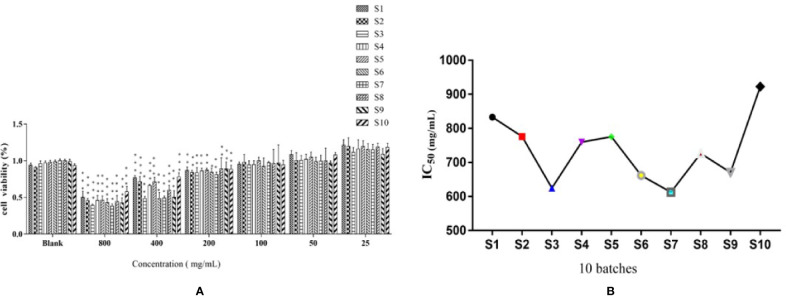
Effects of of *S. heptaphylla* ethanol extracts on the viability of Huh7 cells **(A)** and its IC_50_ values **(B)** *in vitro*. ***P < 0.001, 0.001 < **P < 0.01 vs. Blank group.

In **Figure 2A**, the effects of 10 batches of *S. heptaphylla* ethanol extract on the viability of Huh7 showed when the concentrations of the extracts were 200, 400, 800 μg ·mL^−1^, the 10 batches of samples significantly inhibited the cells viability of Huh7 (*P* < 0.001, 0.001 < *P* < 0.01, *P* < 0.05) in a dose-dependent manner compared with the blank group *in vitro*.

In **Figure 2B**, when the sample concentrations were at 1 g·mL^−1^ (equivalent to the original medicinal material), the IC_50_ values of 10 batches of *S. heptaphylla* on Huh7 cells showed a “W” shape, indicating that at this concentration, the anti-hepatoma effects of *S. heptaphylla* ethanol extract at different collection times were significantly different. The IC_50_ values were the lowest at S3 and S7, indicating that *S. heptaphylla* ethanol extracts had the best anti-liver cancer (Huh7) activity with no significant difference (*P* > 0.05).

### Partial Least Square Regression Analysis

In recent years, spectrum-effect correlation analysis has been successfully applied to the analysis of active components of various traditional Chinese medicine. The main components of spectrum-effect relationship of traditional Chinese medicine include the establishment of fingerprint, pharmacodynamic evaluation and data processing of spectrum-effect correlation (Xu et al., 2014). The most common methods for establishing fingerprints are HPLC, UPLC, GC, and GC-MS (Zhou et al., 2008). Pharmacodynamics often uses experimental models *in vitro* or *in vivo* to obtain “efficacy” information (Zhang et al., 2012). The most common data processing methods are principal component analysis (PCA), canonical correlation analysis (CCA), partial least square analysis (PLSR) and grey correlation analysis (GRDA) (Zhao et al., 2004). Among them, the PLSR method is practical and stable, it can contain all the original fingerprint peaks, and the reaction information is more comprehensive (Alciaturi et al., 2014).

The IC_50_ values of anti-hepatoma activity of samples (equivalent to 1 g ·mL^−1^) were taken as dependent variables and 10 quantitative characteristic chromatographic peak areas as independent variables. The data were averaged by DPS 7.05 statistical software, and then partial least square regression analysis was carried out. After data standardization, the square sum of model error R^2^ increased with the increase of potential factor. When the potential factor reached 6, R^2^ reaches the maximum, and the variance of the explained independent variable is 89.62%.

The regression equation was obtained as follows:

Y=0.000000−0.44285x1+0.183478x2-0.112569x3+0.548834x4-0.183714x5

+0.077159x6−0.245942x7+0.446524x8-0.682518x9+0.884567x10

**Figure 3:**
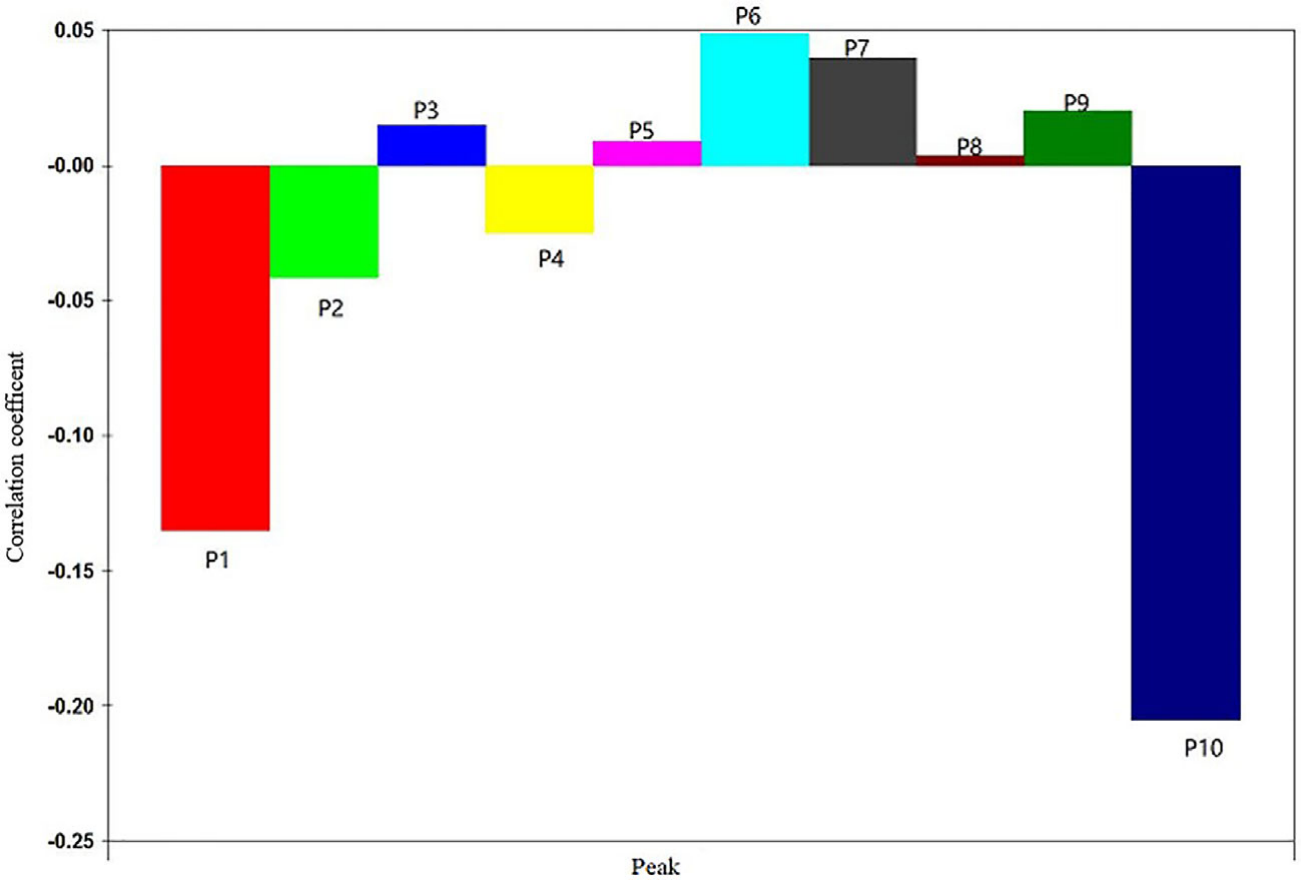
Standardization regression coefficient of PLSR equations of HPLC.

The regression coefficient diagram of the partial least square regression equation was shown in **Figure 3**. P3 and P5, P6, P7, P8, and P9 were positively correlated with the IC_50_ of anti-liver cancer activity of *S. heptaphylla* ethanol extract. However, P1, P2, P4 and P10 were negatively correlated with the anti-hepatoma activity of *S. heptaphylla* ethanol extract. P10 had the strongest anti-liver cancer activity.

### Composition Knock-Out Experiment

The Total Chromatogram of the Sample and the Chromatogram of the Knocked-Out Components

**Figure 4:**
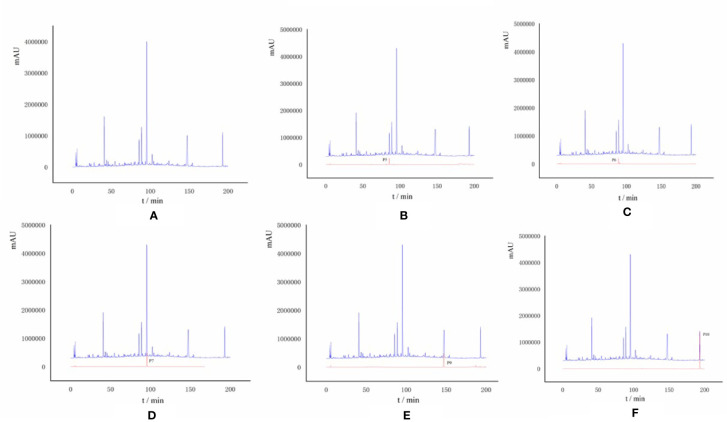
**(A)** HPLC chromatogram of *S. heptaphylla* ethanol extract; **(B–F)** HPLC chromatogram of Knock-Out components.

In **Figure 4A**, the full chromatogram of *S. heptaphylla* ethanol extract at 210 nm wavelength. **Figures 4B–F** are the chromatogram of the target components: P5, P6, P7, P9, and P10, respectively. According to the peak area normalization method, the purity of each target component was more than 85%, and the resolution was good (R > 1.5).

**Figure 5:**
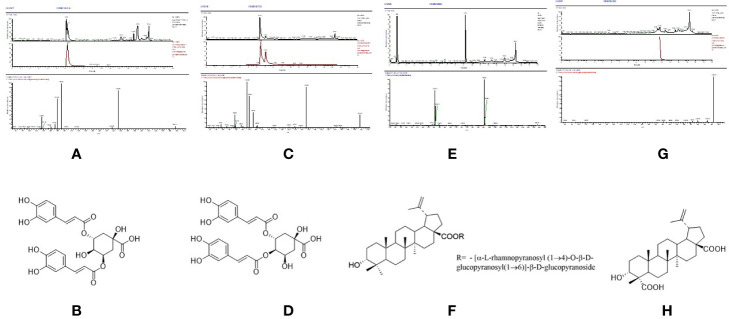
The high resolution mass spectra and chemical structures of Peak 6, 7, 9, 10 **(A–H)** knocked-out components.

P6 (Rt = 89.858 min), the UPLC-MS^2^ results of the knock-out component was shown in **Figure 5A**, in which the molecular formula was C_25_H_24_O_12_. The results of secondary mass spectrometry showed that there were some ion fragments: 515.12/353.09/191.06/173.04/173.04/135.04. According to the reference (Yi, 2017), it was speculated to be 3,5-dicaffeoylquinic acid (**Figure 5B**). Moreover, the retention time of P6 was consistent with that of the reference substance.

P7 (Rt = 96.315 min), the UPLC-MS^2^ results of the knock-out component was shown in **Figure 5C**, in which the molecular formula was C_25_H_24_O_12_. The results of secondary mass spectrometry showed that there were some ion fragments: 515/353/191/179/173/135, 515/335.08/255.07/179.03/155.03/135.04, 515/335.1/317.07/299.06/173.04. According to the reference (Yi, 2017), it was speculated to be 3,4-dicaffeoylquinic acid (**Figure 5D**). In addition, the retention time was consistent with that of the reference substance.

P9 (Rt = 147.446 min), the UPLC-MS^2^ results of the knock-out component was shown in **Figure 5E**, in which the molecular formula was C_48_H_76_O_19_. According to the reference (Adam et al., 1982), it was speculated to be 3*α*-hydroxy-lup-20(29)- ene-23,28-dioic acid 28-*O*-[*α*-l-rhamnopyranosyl (1→4)-*O*-*β*-d-glucopyranosyl (1→ 6)]-*β*-d-glucopyrano-side (**Figure 5F**). In addition, the retention time of P9 was consistent with that of the reference substance.

P10 (Rt = 193.201 min), the UPLC-MS^2^ results of the knock-out component was shown in **Figure 5G**, in which the molecular formula was C_30_H_46_O_5_. The results of secondary mass spectrometry showed that there were some ion fragments: 485.33/467.32/439.32/423.33/, According to the reference (Adam et al., 1982), it was speculated to be 3*α*-hydroxy-lup-20(29)-ene-23,28-dioic acid (**Figure 5H**). Moreover, the retention time was consistent with that of the reference substance.

### Effect of Compound A on the Viability of Normal Hepatocytes and Hepatocellular Carcinoma Cells

**Figure 6:**
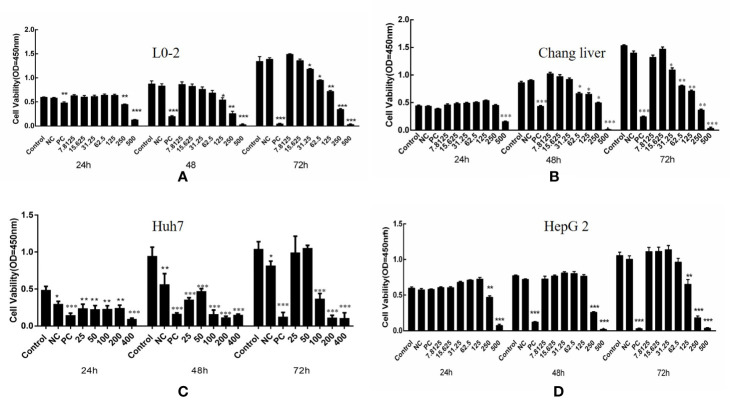
Effects of compound A on the activity of normal hepatocytes and hepatoma cells: L0-2 **(A)**; Chang liver **(B)**; Huh7 **(C)**; HepG2 **(D)**. ***P < 0.001, 0.001 < **P < 0.01, *P < 0.05 vs. control group.

Compound A had the strongest anti-hepatoma activity, the effects of different concentrations of Compound A on the cell viability of normal hepatocyte lines (L0-2, Chang liver) and two hepatoma cell lines (Huh 7, HepG 2) after 24, 48, 72 h were detected by CCK-8 method. In **Figure 6A**, when the concentration of Compound A was higher than 125 nM, and 48 h treatment, Compound A had a slight inhibitory effect on human normal hepatocytes (L0-2), while the positive drugs showed significant cytotoxicity to normal hepatocytes in a time-dependent manner. In **Figure 6B**, after 24 h treatment, when the concentration of Compound A was higher than 500 nM, Compound A had significant cytotoxicity to Chang liver cells, compared with the blank group. After 48 h treatment, when the concentration of Compound A was higher than 62.5 nM, Compound A had significant cytotoxicity. The positive drug had significant cytotoxicity after 48 h. Compared with positive drugs, Compound A had less effect on the viability of normal hepatocytes. In **Figure 6C**, for Huh 7 hepatoma cells, when the concentration of Compound A was higher than 25 nM, it showed significant inhibitory effect on Huh 7 cells in a dose-dependent manner. The values of IC_50_ at 24, 48, and 72 h were 249.9, 285.3, and 199.5 nM, respectively. In **Figure 6D**, Compound A had a significant inhibitory effect on HepG 2 cells when the concentration of Compound A was higher than 25 nM. The values of IC_50_ at 24, 48, and 72 h were 657.7, 315.1, and 186.1 nM, respectively. The results showed that Compound A could significantly inhibit the viability of hepatoma cells, which was consistent with the results predicted by partial least square method. Then, the mechanism of Compound A against hepatoma cells was investigated.

### Effects of Compound A on the Morphology of Two Kinds of Hepatoma Cells

**Figure 7:**
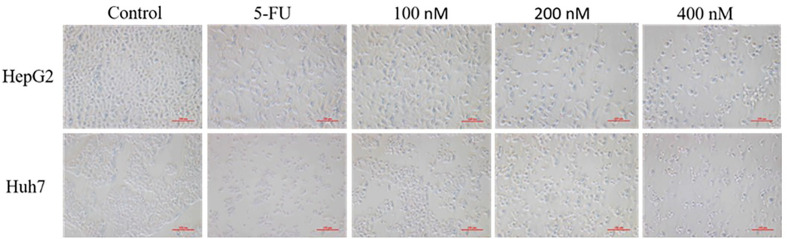
Effects of compound A at different concentrations on the morphology of HepG 2 and Huh 7 cells.

The morphological changes of cells are closely related to the physiological phenomena such as cell proliferation, apoptosis and necrosis. Three concentrations of 100, 200, 400 nM were selected by CCK-8 experiment. HepG 2 and Huh7 hepatoma cells were treated with blank solvent, positive control drug (5-FU) and Compound A (100, 200, 400 nM) for 48 h, and then the morphological changes of HepG 2 and Huh7 hepatoma cells were observed by inverted microscope. Results were shown in **Figure 7**, the cells in the blank group were evenly spread over the bottom of the culture bottle, and the cells were regular, refractive and closely arranged, indicating that the cells were in good condition. While the number of cells in the positive control group and each concentration group of Compound A significantly decreased in a dose-dependent manner, and the cells showed morphological changes such as pseudopodia, deformation and floating.

Thus, the question is whether the inhibitory effects of Compound A on hepatoma cells were achieved by directly causing cell necrosis or by promoting apoptosis? It needs to be further verified.

### Effects of Compound A on Apoptosis of Normal Hepatocytes and Hepatoma Cells

**Figure 8:**
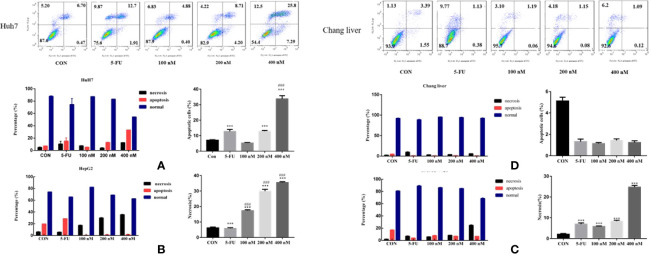
Effects of Compound A on apoptosis of hepatoma cells [**(A)**: Huh 7; **(B)**: HepG 2; **(C)**: SMMC-7721] and normal hepatocytes [**(D)**: Chang liver]. ***P < 0.001 vs. control group; ^###^*P* < 0.001 vs. 5-FU group.

In order to explore the mechanism of Compound A on inhibiting the proliferation of hepatoma cells, the effects of Compound A on apoptosis of normal hepatocytes (Chang liver) and hepatoma cells (Huh 7, HepG 2 and SMMC-7721) were analyzed by Annexin V/PI double staining and flow cytometry. Normal hepatocytes (Chang liver) and hepatoma cells (Huh 7, HepG 2 and SMMC-7721) were treated with blank solvents, positive drug and different concentrations of Compound A for 48 h. In **Figure 8A**, Compound A (200, 400 nM) and positive control group (5-FU) could significantly promote the apoptosis of Huh7 cells (*P* < 0.001), and the effect of Compound A (400 nM) was significantly better than that of positive control (5-FU) (*P* < 0.001). Therefore, we speculated that the inhibitory effect of Compound A on Huh 7 cells might be through promoting apoptosis. In **Figures 8B, C**, Compound A had no significant effects on the apoptosis of other hepatoma cells (HepG 2 and SMMC-7721), but could inhibit the viability of hepatoma cells by directly causing cell necrosis. However, did Compound A also have effects on normal hepatocytes? In **Figure 8D**, Compound A has no significant effect on the apoptosis and necrosis of normal hepatocytes, which further indicated that Compound A not only could inhibit hepatoma cells, but also has less toxic and side effects on normal hepatocytes. Then, we further studied the mechanism of Compound A in promoting apoptosis of Huh 7 cells.

### Mechanism of Compound A on Promoting Apoptosis of Huh 7 Cells

#### Compound A Induces Apoptosis of Huh 7 Cells Through ROS

**Figure 9:**
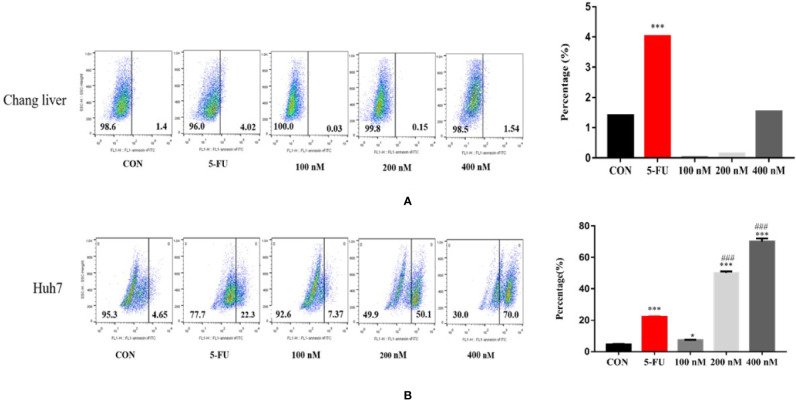
Effects of Compound A at different concentrations on ROS levels in Chang liver **(A)** and Huh 7 **(B)** cells. ***P < 0.001, *P < 0.05 vs. control group; ^###^*P* < 0.001 vs. 5-FU group.

Cancer cells have a variety of apoptotic pathways, and ROS can affect mitochondrial metabolism and induce apoptosis (Masahiko and Masaya, 2013). Therefore, we studied whether Compound A induced apoptosis in Huh 7 cells by changing the level of reactive oxygen species (ROS). In **Figures 9A, B**, flow cytometry analysis showed that both the positive group (5-FU) and Compound A (100, 200, 400 nM) could significantly increase the level of intracellular ROS (*P* < 0.05, *P* < 0.001) compared with the blank group, in addition, the effects of Compound A (200, 400 nM) were significantly stronger than that of the positive control group (5-FU). For the normal hepatocytes (Chang liver), it was found that the positive control group (5-FU) could significantly increase the level of ROS (*P* < 0.001), compared with the blank group, while Compound A had no significant effect on the level of ROS in normal hepatocytes (*P* > 0.001), indicating that Compound A had less cytotoxicity to normal hepatocytes (Chang liver), and significantly lower than positive drugs (5-FU). It has been reported that the increase of intracellular ROS could induce the activation of mitochondrial apoptosis pathway. Then, the next study focused on the changes in the content of mitochondrial apoptotic proteins.

#### Regulatory Effect of Compound A on the Expression of Apoptosis-Related Proteins in Huh 7 Cells

**Figure 10:**
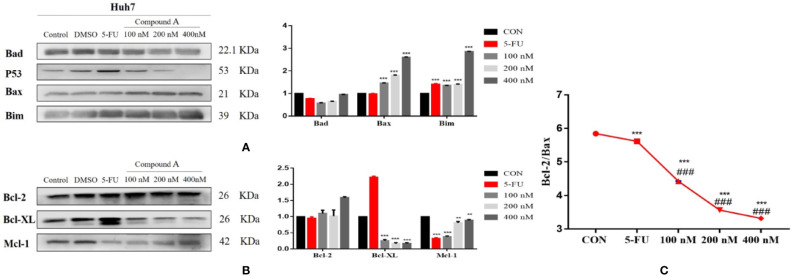
The regulatory effect of Compound A on the expression of apoptosis related proteins in Huh7 cells. **(A)** pro-apoptotic protein; **(B)** anti-apoptotic protein; **(C)** ratio of Bcl-2 to Bax. ***P < 0.001,**P < 0.01 vs. control group; ^###^*P* < 0.001 vs. 5-FU group.

The increase of intracellular ROS level can induce the activation of mitochondrial apoptosis pathway. In order to further explore the mechanism of Compound A-induced apoptosis in Huh 7 cells, the expressions of Bcl-2 family proteins were detected. The change in the content of pro-apoptotic proteins was shown in **Figure 10A**, Compound A (100, 200, 400 nM) could significantly increase the content of mitochondrial pro-apoptotic proteins Bax and Bim (*P* < 0.001) compared with the blank group, and the effects were better than that of positive drug (5-FU). As for the anti-apoptotic protein, in **Figure 10B**, Compound A (100, 200, 400 nM) could significantly decrease the contents of Bcl-xL and Mcl-1 (*P* < 0.001), with no significant effect on the content of Bcl-2 (*P* > 0.001) compared with the blank group. Because the ratio of Bcl-2 to Bax determines the permeability of mitochondrial membrane, when the ratio is low, the permeability of mitochondrial membrane increases and apoptotic factors are transferred from mitochondria to the nucleus, which in turn induces cell apoptosis. On the contrary, it is difficult for apoptotic factors to transfer to the nucleus. In **Figure 10C**, Compound A (100, 200, 400 nM) and the positive control group (5-FU) significantly decreased the proportion of Bcl-2/Bax protein compared with the blank group (*P* < 0.001), and the effects of Compound A (100, 200, 400 nM) were significantly better than that of the positive control group (5-FU). It was inferred that Compound A might induce apoptosis of Huh 7 cells by regulating the expression of mitochondrial apoptotic proteins.

## Materials and Methods

### General

Shimadzu High-Performance Liquid Chromatograph (Japan Shimadzu Co., Ltd). Thermo Ultimate 3000 UHPLC, Q Exactive (Thermo Scientific, US). chromatographic column Purospher^®^ STAR LP RP-18 (Merck KGaA, Germany). MultiskanMK3 Full wavelength enzyme marker (Thermo Electron, US). GRP-9270 Constant temperature incubator (Sen xin Experimental instrument Co., Ltd, Shanghai). AB135-S Electronic balance (Mettler-Toledo Instruments Co., Ltd., Switzerland). Rotary evaporation meter (Tokyo physical and Chemical Instruments Co., Ltd.), Centrifuge precipitator (Shanghai Surgical Instruments Factory). Flow cytometry (Becton Dickinson, BD, US). Multicolor fluorescence, chemiluminescence and visible light imager (ProteinsiMpleFluorcheM Q). Electrophoretic apparatus (EPS600 Tanon); Inverted microscope (Nikon ECLiPSETS100).

### Drugs and Reagents

Acetonitrile was purchased from Fisher (HPLC, US); pure water was obtained from Wahaha Group (Hangzhou); Dulbecco’s modified eagle medium (DMEM), Penicillin and streptomycin, Penicillin and streptomycin and PVDF membrane were purchased from Beijing Solarbio Science & Technology Co., Ltd. non-fat milk powder, Trypsin, EDTA, CCK-8 Kit, 6/96 well cell culture plate, Annexin V-FITC/Pl, Bax、Bcl-2、BID、Bcl-XL、Bak、Bim、β-actin and corresponding antibody were purchased from Becton Dickinson (BD, US); BCA protein quantitative Kit and Acrylamide were obtained from biyuntian biological Co., Ltd (Shanghai); fetal bovine serum (FBS) was purchased from Gibco (Grand Isoland, US); Thiazolyl blue(MTT) was purchased from Hualan Chemical Technology Co., Ltd (Shanghai).

### Plant Material

10 batches of *S. heptaphylla* leaves were collected from the foothills of Qingyuan Mountain in Quanzhou City, Fujian Province, as detailed in **Table 3**. All the samples were identified as the leaves of *S. heptaphylla* by Professor Changqin Li (Henan University). The samples now were stored in the Medical College of Huaqiao University and the National R & D Center for Edible Fungus Processing Technology, Henan University.

**Table 3:** Collection of *S. heptaphylla* leaves.

NO.	Sample origin	Collection time
S1	Qingyuan Mountain, Quanzhou City (118°36’ E, 24°58 ‘ N)	2017.03.15
S2	Qingyuan Mountain, Quanzhou City (118°36’ E, 24°58 ‘ N)	2017.04.15
S3	Qingyuan Mountain, Quanzhou City (118°36’ E, 24°58 ‘ N)	2017.05.15
S4	Qingyuan Mountain, Quanzhou City (118°36’ E, 24°58 ‘ N)	2017.06.15
S5	Qingyuan Mountain, Quanzhou City (118°36’ E, 24°58 ‘ N)	2017.07.15
S6	Qingyuan Mountain, Quanzhou City (118°36’ E, 24°58 ‘ N)	2017.08.15
S7	Qingyuan Mountain, Quanzhou City (118°36’ E, 24°58 ‘ N)	2017.09.15
S8	Qingyuan Mountain, Quanzhou City (118°36’ E, 24°58 ‘ N)	2017.10.15
S9	Qingyuan Mountain, Quanzhou City (118°36’ E, 24°58 ‘ N)	2017.11.15
S10	Qingyuan Mountain, Quanzhou City (118°36’ E, 24°58 ‘ N)	2018.12.15

### Experimental Methods

#### Sample Solution Preparation

10 batches of *S. heptaphylla* leaves were crushed (40 mesh), about 5.0 g, 10 times the amount of 70% ethanol, cold soaked for 3 times, each time for 3 days, combined with filtrate, decompressed and concentrated to obtain the total extract of *S. heptaphylla* leaves. The 200.00 mg extract was precisely weighed and dissolved in 10 mL 70% ethanol and filtered with a 0.22-μm micro porous membrane to prepare a solution, equivalent to 1 g· mL^−1^ of the original medicinal material.

#### Conditions for Fingerprint Analysis

Chromatographic conditions: RP-18 endcapped column (4.6 mm × 250 mm, 5 μm); mobile phase was acetonitrile (B)-0.1% phosphate water (D). The elution procedure was shown in **Table 4**. The flow rate was set at 0.6 mL/min, and the column temperature was 25 °C. The detection wavelength was set at 210 nm with the injection volume of 5 μL.

**Table 4:** Elution procedure.

Time (min)	B (%)	D (%)
0–50	5%–15%	95%–85%
50–100	15%–25%	85%–75%
100–135	25%–33%	75%–67%
135–145	33%–35%	67%–65%
145–165	35%–41%	65%–59%
165–200	41%–90%	59%–10%

#### The Method of Knocking Out the Target Components

Under the optimal conditions of high performance liquid chromatography (HPLC), the 70% ethanol extract (equivalent to 1 g· mL^−1^) was injected into 5 μL, and the chromatogram at 210 nm wavelength was recorded. According to the spectrum-effect relationship, the retention time of the chromatographic peaks related to the anti-hepatoma activity was analyzed, and the eluents of the corresponding peaks were collected and concentrated under reduced pressure to get the target compounds. Each target peak was prepared 10 times in liquid phase, and concentrated the eluate respectively. Dissolved with 0.3 mL 70% ethanol solution and passed through 0.22 μm microporous membrane to obtain the sample containing the target peak (marked as Px).

#### Mass Spectrometry Analysis

Chromatographic conditions: Watres BEHC-18 column (2.1 × 50 mm, 1.7 μm), mobile phase was acetonitrile (A)-0.1% formic acid aqueous solution (B), The elution procedure was follows: 0–5 min, (A)10%–(B) 90%; 5–30 min, (A) 95%–(B) 5%; 30 to 55 min, (A) 95% to (B) 5%; 55 to 56 min, (A) 10%–(B) 90%; 56–61 min, (A) 10%–(B) 90%. The flow rate was set at 0.3 mL/min, and injection 0.2 μL, determination.

Mass spectrometry conditions: sheath gas flow rate: 35 arb; auxiliary gas flow rate: 10 arb; spray voltage: 3.5 kV; capillary temperature: 320 °C, polarity: positive; full scan parameters: resolution 7000; AGC target: 3e6; Maximum IT: 100 ms; scanning range: 50-800 *m/z*. Second-level scanning parameters: resolution: 17500; AGC target: 1e5; Maximum IT: 50 ms; isolation window: 4.0 *m/z*; collision energy: 30.

#### Cell Culture

L0-2, Chang liver, HepG 2, SMMC-7721 and Huh 7 cells were cultured in the DMEM medium containing 10% FBS, 1% penicillin and streptomycin mixture and cultured in the incubator at 37 °C and 5% CO_2_. Take logarithmic growth phase cells for follow-up experiment.

#### Microscopic Observation of Cell Morphology

After the cells were treated with different concentrations of drugs for 48 h, the cells were collected and randomly taken 5 visual fields under an inverted microscope to take pictures (20 × objective lens).

#### Cell Viability Detection

##### MTT

The cells of logarithmic growth phase were inoculated in 96-well plates (1 × 10^4^ cells/well), and incubated in incubator at 37 °C and 5% CO_2_ for 24 h. Thereafter, the supernatant was discarded by liquid transfer gun. The ethanol extracts of *S. octophylla* were diluted from 800 μg/mL to 800, 400, 200, 100, 50 and 25 μg/mL, respectively, and the blank control group was given DMEM medium with 6 compound holes in each concentration. Then incubated in 37 °C incubator for 24 h, 10 μL MTT solution was added to each well, then the supernatant was discarded after 4 h, and then DMSO solution was added to dissolve it. The absorbance was measured at 490 nm wavelength for 10 min.

##### CCK-8

Follow the operation steps of the CCK-8 test kit instructions. The logarithmic growth phase cells were inoculated into 96-well plates (2 × 10^3^ cells/well). 12 h later, Compound A of different concentrations were added, and 6 compound holes were set up for each concentration. The cells were cultured at 37 °C for 24, 48 and 72 h, then the supernatant was discarded and washed with PBS twice. Then, 10% CCK-8 solution was added and cultured at 37 °C for 2 h. The absorbance was measured by enzyme labeling instrument at 450 nm wavelength.

#### Flow Cytometry Analysis

The apoptosis of related cells was detected by FITC Annexin V apoptosis detection kit. The cells in logarithmic growth phase were inoculated in 6-well plates (1 × 10^6^ cells/well) and cultured for 24 h. The original culture medium was abandoned and Compound A (100, 200, 400 nM) solution was added. The blank control group was DMSO, positive control group was 5-fluorouracil (5-FU). Each group was provided with 3 multiple holes and incubated in an incubator at 37 °C and 5% CO_2_ for 48 h. After discarding the original culture medium and washing with PBS for 3 times, the cells were digested with 0.25% trypsin, centrifuged for 5 min, washed twice with 1 mL precooled PBS, re-suspended in 200 μL binding buffer, then mixed with 10 μL Annexin V-FITC, 15 min, and 300 μL binding buffer were added away from light at room temperature, finally, 5 μL PI was added away from light, staining for 15 min to determine the apoptosis rate of each sample by flow cytometry. The left upper quadrant (Q1) was necrotic cells, the right upper quadrant (Q2) was late apoptotic cells, the right lower quadrant (Q3) was early apoptotic cells, and the left lower quadrant (Q4) was normal cells. Results the average percentage of apoptotic cells was analyzed by FlowJo software.

#### Detection of the Expression of Related Proteins in Apoptosis Signal Pathway by Western Blot

The cells in logarithmic growth phase were evenly inoculated in 6-well plates (1 × 10^6^ cells/well), and then treated with Compound A (20, 50, 100 nM), blank control group (DMSO) and positive control group (5-FU) for 48 h in an incubator at 37 °C and 5% CO_2_. Then, cells were harvested and lysed on ice for 30 min in Radio Immunoprecipitation Assay (RIPA), and centrifuged at 4 °C, 12000 rpm for 20 min, to absorb the supernatant and transfer it to an EP tube. The protein concentration was measured by BCA protein concentration assay kit. Cell lysates were then loaded onto 10% SDS-PAGE for analysis of Bax、Bcl-2、BID、Bcl-XL、Bak、Bim, and β-actin. Proteins were transferred onto a polyvinylidene fluoride membranes (PVDF), which were blocked in 5% non-fat milk in Tris-buffered saline with 1% Tween 20 for 2 h. Then, the PVDF membrane was incubated with the primary antibody overnight at 4 °C. The membranes were washed thoroughly and incubated with horseradish peroxidase-conjugated secondary antibodies. After washing, protein bands were visualized using enhanced chemiluminescence (ECL) and use Imag J software to analyze the gray value of the protein.

### Statistical Analysis

The partial least square regression analysis method was used to analyze the data of 10 quantitative characteristic maps and the anti-hepatoma activities of 10 batches *S. heptaphylla* extracts. The retention time of each peak of the characteristic spectrum of *S. heptaphylla* was corrected by using the software “similarity Evaluation system of chromatographic fingerprint of traditional Chinese Medicine version 1.0 A” recommended by Chinese Pharmacopoeia Committee, and the peak area was averaged. The data of 10 quantitative characteristic peaks were obtained. Using DPS 7.05 software to analyze the area of 10 quantitative characteristic peaks as independent variables, the IC_50_ values of (X), 10 batches of *S. heptaphylla* extract against liver cancer as dependent variables (Y), the partial least square regression equation was established, and the correlation between quantitative characteristic peaks and anti-hepatoma activity was screened.

The results were expressed by arithmetic mean and standard deviation, and the significant differences were compared by SPSS software single factor analysis of variance (One-Way ANOVA). All the pictures were drawn with Graphpad Prism 7.0.

## Discussion

“Spectrum-Effect Relationship” and “Component Knock-Out” technology has been successfully applied in screening the active ingredients of natural products (Shi et al., 2018 and Li et al., 2019). In this paper, partial least square method was used to analyze the correlation between 10 quantitative characteristic chromatographic peaks of *S. heptaphylla* and the anti-hepatoma activity (IC_50_ value) of *S. heptaphylla*. It was inferred that P10 had the strongest anti-liver cancer activity by spectral correlation analysis. Bioactive components of natural products can be isolated quickly and efficiently by using “component knock-out” technology, which has advantages of accuracy and efficiency. In this manuscript, based on spectral correlation analysis, P10 with anti-hepatoma activity was prepared rapidly by component knock technique, and P10 was determined as 3*α*-hydroxy-lup-20(29)- ene-23,28-dioic acid (Compound A) by “Component knock-out” method and high resolution mass spectrometry. HPLC comparison showed that the results were consistent with reference standard.

In this manuscript, the results of CCK-8 test showed that Compound A could significantly inhibit the viability of hepatoma cells Huh 7, HepG 2 and SMMC-7721, which was consistent with the results speculated by spectral correlation analysis. Compound A could inhibit the viability of liver cancer cells by directly necrotizing tumor cells or indirectly inducing tumor cell apoptosis? The morphological changes of cells are often closely related to necrosis, apoptosis and so on. We found that Huh7 cells showed typical apoptotic morphology, which was further verified. The apoptosis of three kinds of hepatocellular carcinoma cells and normal hepatocytes were analyzed by flow cytometry. It was found that the apoptotic proportion of Huh7 cells significantly increased after Compound A treatment, while the necrotic proportion of HepG 2 and SMMC-7721 cells increased significantly, but there was no significant effect on normal hepatocytes.

The classical cytological theory points out that apoptosis is a spontaneous way of cell death, which mainly includes exogenous and endogenous apoptosis pathways (Daugas, 2001; Alciaturi et al., 2014). Including mitochondrial apoptosis pathway, death receptor apoptosis pathway and endoplasmic reticulum apoptosis pathway (Fulda and Debatin, 2006). Among them, mitochondrial apoptosis pathway is the most common one (Ashkenazi and Salvesen, 2014), which is regulated by Bcl-2 protein family, Caspase family and other key factors. Among them, Bcl-2 family regulates the permeability of mitochondrial inner and outer membrane, thus affecting the process of cell apoptosis (Martinou and Youle, 2011). The Bcl-2 protein family is mainly composed of anti-apoptotic proteins and pro-apoptotic proteins (Tsukahara et al., 2006). Anti-apoptotic proteins include Bcl-xl, Mcl-l and so on, in which Bcl-2 protein is the main sensor of apoptosis (Hockenbery et al., 1990; Boise et al., 1993). Anti-apoptotic proteins can stabilize the mitochondrial membrane, prevent the release of many kinds of mitochondrial apoptotic proteins such as Cyt-c, and prevent the further activation of Caspase (Kutuk and Basaga, 2006). Pro-apoptotic proteins, including Bax, Bak and Bad, could change the mitochondrial membrane potential and enhance the mitochondrial permeability. Cytochrome C (Cyt-c) is released from the mitochondria into the cytoplasm and further binds with apoptosis-related factors (Apaf-1) to form oligomers, which binds Pro-Caspase-9 to form apoptotic bodies to activate Caspase-9, and then activate Caspase-3, to lead cell apoptosis. In addition, the ratio of Bax to Bcl-2 protein may play a key role in the process of apoptosis (Liang and Cao, 2014). When the proportion is low, the permeability of the mitochondrial membrane increases, and the apoptotic factor is transferred from the mitochondria to the nucleus, which could induce the apoptosis. On the contrary, it is difficult to transfer the apoptotic factor to the nucleus. Intracellular ROS could affect mitochondrial metabolism (Masahiko and Masaya, 2013) and then activate the pathway of mitochondrial apoptosis.

It is reported that lupine pentacyclic triterpenes have strong and broad-spectrum anti-tumor activity, low toxicity and selectivity to tumor cells, and their mechanism is independent of p53 pathway, so they are expected to become excellent anti-tumor lead compounds (Wang et al., 2017). Betulinic acid can inhibit the proliferation of HepG 2 cells through Wnt pathway. Oleanolic acid inhibits proliferation and induces apoptosis of HepG 2 cells by regulating ROS and MMP (Miao et al., 2016). In addition, betulinic acid, 23-hydroxybetulinic acid, betulin and lupeol have significant anti-cancer activities through activating mitochondrial apoptosis pathway, and regulate the levels of Bcl-2 and ROS (Wang et al., 2017).

In order to further explore the mechanism of Compound A in promoting apoptosis of Huh7 cells. We detected the changes of apoptosis-related proteins of Bcl-2 family proteins and ROS in Huh7 cells. The results showed that Compound A (100, 200, 400 nM) could significantly increase the content of mitochondrial pro-apoptotic proteins Bax and Bim (*P* < 0.001), and its effect was better than that of positive drugs (5-FU). As for anti-apoptotic protein, Compound A (100, 200, 400 nM) could significantly reduce the content of Bcl-xL and Mcl-1 (*P* < 0.001), compared with the blank group, and Compound A (100, 200, 400 nM) and 5-FU could significantly reduce the proportion of Bcl-2/Bax protein (*P* < 0.001).

## Conclusion

Spectrum Effect Relationship and Component Knock-Out were the rapid method to infer anti-hepatoma activity of compound in *S. heptaphylla* leaves and Compound A was determined to be one of the main anti-hepatoma active components. Compound A could significantly inhibit the proliferation of all kinds of hepatoma cells (Huh7, HepG2 and SMMC-7721), and had little toxicity to normal hepatocytes. It could promote the apoptosis of Huh7 cells by up-regulating the level of ROS in Huh7 cells, activating intracellular mitochondrial apoptosis pathway and regulating the level of Bcl-2 family proteins in mitochondrial apoptosis pathway.

## Data Availability Statement

All datasets presented in this study are included in the article/supplementary material.

## Author Contributions

WK and YZ conceived the research subject. XL, NJ, XX, and CL conducted the experiments, collected the plant specimens, separated and analysis compounds, analyzed and interpreted the data, as well as prepared the first draft. WK and XL critically read and revised the paper. All authors contributed to the article and approved the submitted version.

## Funding

This work was supported by Key Project in Science and Technology of Henan Province (192102110112).

## Conflict of Interest

The authors declare that the research was conducted in the absence of any commercial or financial relationships that could be construed as a potential conflict of interest.

## Abbreviations

ROS, reactive oxygen species; HPLC, high-performance liquid chromatography; CCK-8, cell counting kit-8; IC_50_, half-inhibitory concentration; DMSO, dimethyl sulfoxide; 5-FU, 5-fluorouracil; IRPA, radio immunoprecipitation assay; PVDF, polyvinylidene fluoride membranes; MTT, 3-(4,5)-dimethylthiahiazo (-z-y1)-3,5-di- phenytetrazolium bromide; DMEM, Dulbecco’s modified eagle medium; TCM, Traditional Chinese Medicine; ECL, enhanced chemiluminescence; Compound A, 3*α*-hydroxy-lup-20(29)-ene-23,28-dioic acid; HCC, hepatocellular carcinoma; FBS, fetal bovine serum.
